# Chemical Composition and Biological Activities of the Essential Oils from Different Parts of *Rosa bracteata* J.C.Wendl

**DOI:** 10.3390/molecules30194021

**Published:** 2025-10-08

**Authors:** Shiyu Song, Yifang Chen, Hongrui Chen, Qinglei Han, Pengxiang Lai

**Affiliations:** SDU-ANU Joint Science College, Shandong University, Weihai 264209, China

**Keywords:** *Rosa bracteata*, essential oil, antibacterial, synergistic, cytotoxic, antioxidant

## Abstract

*Rosa bracteata* J.C.Wendl. is a thorny, clump-forming or trailing perennial evergreen shrub native to China. The current analysis was designed to explore the chemical constituents and determine the in vitro antimicrobial, cytotoxic, and antioxidant properties of the essential oils (EOs) of the stems, leaves, and flowers of *Rosa bracteata* for the first time. The chemical composition of the essential oils obtained through hydro-distillation was characterized by means of gas chromatography–mass spectrometry (GC–MS) and gas chromatography with a flame ionization detector (GC–FID). Thirty-seven, thirty-six, and forty-two constituents were identified from leaf oil (LEO), stem oil (SEO), and flower oil (FEO), representing 96.3%, 95.9%, and 97.4% of the total oil constituents, respectively. The LEO was mainly composed of 1-pentadecene, *α*-cadinol, and hexadecanoic acid. However, the main identified components of SEO were (*E*)-nerolidol, phytol, and benzyl benzoate, and the main components of the flower oil were ethyl octanoate, octanoic acid, and α-cadinol. All of the EOs exhibited antibacterial activities against both Gram-positive and Gram-negative bacteria with MIC values ranging from 40.00 to 640.00 μg/mL. In addition, the checkerboard method demonstrates potent synergistic effects of *Rosa bracteata* EOs when combined with commercial antibiotics (chloramphenicol and streptomycin). In the MTT test, SEO (IC_50_: 37.91 ± 2.10 to 51.15 ± 6.42 μg/mL) showed stronger cytotoxic activity against four cancer cell lines (MCF-7, A549, HepG2, and HCT-116) during the incubation time of 48 h in comparison to the EOs isolated from the other plant parts. Overall, these findings reveal the chemical composition and significant bioactivity of *R. bracteata* EOs for the first time, suggesting their potential as promising natural agents for therapeutic applications, especially in combination therapies to combat antibiotic resistance.

## 1. Introduction

Essential oils (EOs), which are complex mixtures of volatile compounds derived from plants, have been valued since antiquity for their medicinal and preservative properties [1,2,3,4,5]. These secondary metabolites present in various plant parts, comprising terpenes, phenolics, and their derivatives, are responsible for a wide spectrum of biological activities, including potent antimicrobial and antioxidant effects [6,7,8,9]. Modern research is now focused on validating and harnessing these activities, positioning EOs as promising natural alternatives to synthetic compounds in healthcare and agriculture [10].

The genus *Rosa* (Rosaceae), comprising over 200 species and countless cultivars, is one of the world’s most iconic and economically important plant groups, revered for its ornamental beauty, cultural significance, and diverse applications in perfumery, food, and traditional medicine [11,12,13]. Beyond their aesthetic value, rose flowers and hips are rich sources of bioactive compounds like vitamin C, polyphenols, and galactolipids, contributing to their use in treating inflammation, pain, and gastrointestinal disorders [14,15]. Phytochemical research on the genus has extensively focused on the non-volatile components of rose hips and petals, as well as the essential oil of renowned species like *R. damascena* and *R. centifolia*, which are prized in the fragrance industry for their rich concentrations of monoterpene alcohols like citronellol, geraniol, and nerol [16,17]. Rose essential oil is a valuable aromatic product; however, its chemical composition varies considerably across different rose species [18]. The major compounds reported in rose oils are *β*-citronellol, nonadecane, geraniol, heneicosane, and eugenol [19,20,21,22,23]. These compounds, such as geraniol and eugenol, are not only responsible for the rose’s ecological function and unparalleled status in perfumery but also confer a wide spectrum of biological activities, including antimicrobial, antioxidant, anti-inflammatory, analgesic, and anti-anxiety effects [24,25,26].

Among this well-known genus, *Rosa bracteata* J.C.Wendl., commonly known as the Macartney rose, presents a particularly interesting and understudied subject. Native to southern China, this vigorous, evergreen climbing rose is distinguished by its large, solitary white flowers subtended by prominent, feathery bracts—a characteristic from which its species name is derived [27]. It is resistant to common rose diseases like black spot, and its roots and leaves have been used in folk medicine as astringents and for treating diarrhea, suggesting a potent phytochemical profile [28]. Initial phytochemical studies on the fruits of *Rosa bracteata* have revealed a unique profile, isolating six compounds and identifying four of them. Significantly, they documented the first-ever occurrence of 5-hydroxymethyl furfural and 5-acetoxymethyl furfural in the genus *Rosa* L., alongside the known compounds beta-sitosterol and oleanolic acid [28].

However, in contrast to the well-documented volatile profiles of commercial rose species, the essential oil of *R. bracteata* remains unexplored. Preliminary analyses suggest a unique chemical signature differing significantly from the classic “rose-scented” monoterpene profile [26,29,30]. This distinct chemical composition suggests the presence of biological activities that have not been investigated. Therefore, a comprehensive analysis of the essential oils from different plant parts of *R. bracteata*—along with an evaluation of their associated antioxidant, antimicrobial, cytotoxic, and synergistic properties—is crucial to fully understand its phytochemical value and potential applications. This study aims to fill this research gap by providing comparative phytochemical and pharmacological properties of the leaf, stem, and flower essential oils of *Rosa bracteata*, thereby demonstrating this species as a novel source of bioactive volatile compounds within the *Rosa* genus.

## 2. Results and Discussion

### 2.1. Analysis of Chemical Composition

**Figure 1:**
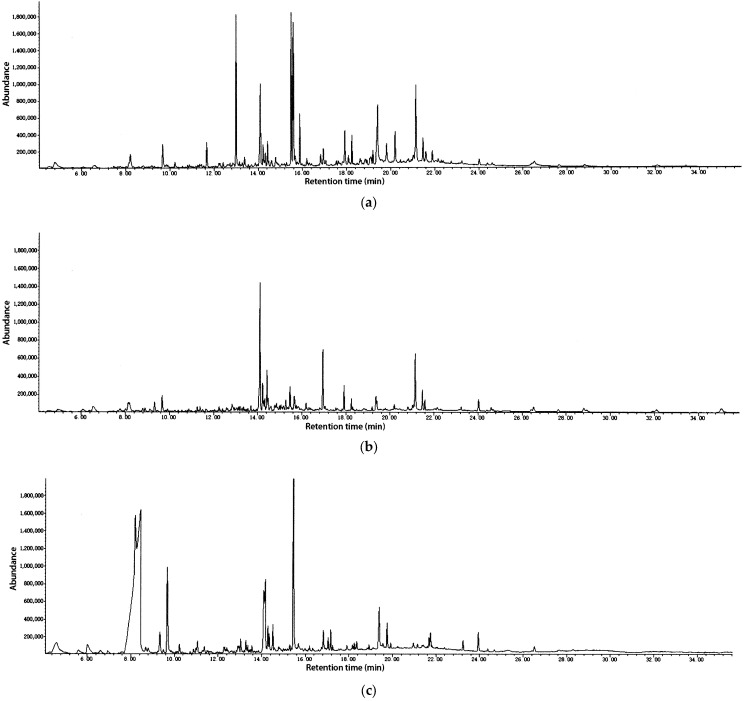
Chromatogram of GC–MS analysis of the essential oil of (**a**) *R. bracteata* leaves; (**b**) *R. bracteata* stems; (**c**) *R. bracteata* flowers.

The hydrodistillation of different parts of *Rosa bracteata* yielded three essential oils characterized by a typical odor, with yields for leaves, stems, and flowers of 0.12%, 0.06%, and 0.08% (dry weight), respectively. The analysis of three hydrodistilled essential oils from different parts of *R. bracteata* by GC–FID and GC–MS revealed 37, 36, and 42 various compounds (Table 1), representing 96.3%, 95.9%, and 97.4% of total oils in leaf, stem, and flower samples, respectively. The corresponding chromatogram is depicted in Figure 1a–c). The primary chemical constituents in the leaf oil (LEO) were (*Z*)-7-tetradecen-1-ol (13.9%), 1-pentadecene (10.6%), *α*-cadinol (10.4%), hexadecanoic acid (8.0%), (*E*)-nerolidol (7.1%), and phytol (6.9%). (*E*)-Nerolidol (18.5%), phytol (9.1%), benzyl benzoate (9.0%), (2*E*)-2-hexenyl benzoate (5.5%), and *α*-cadinol (4.9%) were the main compounds in the stem oil (SEO). For the flower essential oil (FEO) of *R. bracteata*, the main compounds were found to be ethyl octanoate (31.4%), octanoic acid (25.8%), *α*-cadinol (10.4%), and dodecanoic acid (4.4%). The results showed an almost entirely different composition in the flower oil compared to the leaves and stems. The contents of ethyl octanoate and octanoic acid in the flower oil reached 31.4% and 25.8%, respectively, whereas these two compounds were not found in the leaves and stems. The fact that ethyl octanoate can give off an aroma may be the reason for the higher content of ethyl octanoate in the flowers [31]. The fragrance and value of traditional rose oils are defined by a suite of volatile monoterpene alcohols and phenethyl alcohol, including citronellol, geraniol, and nerol. These compounds are dominant in the essential oils of *R. damascena* (Damask rose) and *R. centifolia* (Cabbage rose), which are primarily cultivated for the perfume industry [17,32,33,34,35]. In contrast, the essential oils of *Rosa bracteata* analyzed in this study are either devoid of or contain only trace amounts of these characteristic “rose scent” monoterpenes. *Rosa bracteata* essential oils are not “typical” rose oils but are instead characterized by a high abundance of bioactive compounds. This makes *R. bracteata* a particularly interesting species for pharmacological research.

### 2.2. Evaluation of Antibacterial Activity

The antibacterial effects of *R. bracteata* EOs against Gram-positive and Gram-negative bacterial strains were evaluated using microdilution assays with chloramphenicol as a reference. The results demonstrate that all three essential oils possess notable antibacterial properties (Table 2). However, the stem essential oil (SEO) consistently exhibited the strongest inhibitory activity, showing the lowest minimum inhibitory concentration (MIC) values (40.00 to 160.00 μg/mL) against three of the four tested strains (*Bacillus subtilis*, *Staphylococcus aureus*, and *Escherichia coli*). For *Pseudomonas aeruginosa*, SEO was as effective as LEO (MIC of 320.00 μg/mL). The minimum bactericidal concentration (MBC) values of *R. bracteata* EOs were often equal to or one dilution higher than the MIC values, indicating a bactericidal effect. In contrast, against *P. aeruginosa*, the MBC for SEO was 1280.00 μg/mL, which is four times its MIC, suggesting a less potent bactericidal effect against this resilient bacterium. The flower essential oil (FEO) consistently showed the weakest activity against the Gram-negative strains, with the highest MIC and MBC values.

The superior antibacterial efficacy of SEO is particularly noteworthy. Its potency, especially against the Gram-positive bacteria *B. subtilis* and *S. aureus* (MIC = 40.00 and 80.00 μg/mL, respectively), can be attributed to its major chemical compounds. SEO contains a powerful combination of known antimicrobial terpenes: (*E*)-nerolidol, phytol, and α-cadinol [39,40,41,42,43,44,45]. In addition, benzyl benzoate is a well-documented antimicrobial agent with proven efficacy against a range of microbes [46]. Its presence in SEO, in synergy with the membrane-disrupting terpenes (nerolidol and phytol), likely creates a more potent antibacterial blend than what is found in LEO. This synergy could enhance the disruption of the bacterial cell membrane, a primary mechanism of action for many essential oil components [47]. The lower activity of FEO aligns with its chemical composition, which is dominated by esters (ethyl octanoate) and fatty acids (octanoic acid and dodecanoic acid). While some fatty acids exhibit antimicrobial properties, they are typically less potent than the complex terpenoid cocktails present in the leaf and stem oils [48,49,50,51].

### 2.3. Evaluation of Synergistic Interactions

The synergistic antibacterial effects between the essential oils from *Rosa bracteata* (LEO, SEO, and FEO) and conventional antibiotics (chloramphenicol and streptomycin) were evaluated using the checkerboard method against two Gram-positive and two Gram-negative bacterial strains. The interaction was quantified using the Fractional Inhibitory Concentration Index (FICI), where FICI ≤ 0.5 indicates synergy (S), >0.5 to ≤4 indicates additivity/indifference, and >4 indicates antagonism [52]. The results are summarized in Table 3 and Table 4. All combinations of essential oils and antibiotics resulted in synergistic (FICI ≤ 0.5) interactions against all tested bacterial strains. The most potent synergy (lowest FICI values, often ≤0.10) was consistently observed against the Gram-negative bacteria *E. coli* and *P. aeruginosa*. The stem essential oil (SEO) consistently yielded the lowest FICI values (strongest synergy) in almost every combination, particularly with streptomycin. Notably, the flower essential oil (FEO), which demonstrated the weakest direct antibacterial activity on its own, showed remarkably strong synergy, especially with antibiotics against the resilient *P. aeruginosa*. The results demonstrate a potent synergistic interaction between the essential oils of *Rosa bracteata* and two distinct classes of antibiotics. This finding is of significant practical importance, as it suggests these oils could be used as potent adjuvant therapy to enhance the efficacy of conventional antibiotics, potentially overcoming resistance and reducing required dosages.

The most noteworthy result is the remarkable synergy observed against the Gram-negative bacteria *E. coli* and particularly *P. aeruginosa*. *P. aeruginosa* is renowned for its intrinsic resistance to antibiotics due to its low-permeability outer membrane and efficient efflux pumps [53,54,55]. The fact that all tested essential oils, including the less antibacterial FEO, significantly enhanced the efficacy of antibiotics against this pathogen, indicating a highly effective mechanism. The probable explanation is that the hydrophobic components of the essential oils disrupt the integrity of the outer membrane of Gram-negative bacteria. By damaging this critical barrier, the oils are likely to increase membrane permeability, facilitating the influx of antibiotics like chloramphenicol and streptomycin into the bacterial cell, thereby overcoming the primary mechanism of resistance [56]. This mechanism is less critical in Gram-positive bacteria, which lack an outer membrane, which is why the synergy, while still present, is not as dramatically potent (i.e., FICI values are higher).

The remarkable synergistic ability of the flower essential oil (FEO) is an interesting discovery. While FEO’s primary constituents (ethyl octanoate, octanoic acid, and dodecanoic acid) are not potent antibacterial agents on their own, they appear to be highly effective membrane permeabilizers. Medium-chain fatty acids and their esters are known to integrate into and disrupt lipid bilayers [51]. This action appears to be sufficient to “open the gates” for antibiotics, especially against the tough outer membrane of *P. aeruginosa*, making FEO a highly effective synergistic agent despite its weak standalone activity.

This study reveals that essential oils from *Rosa bracteata* are not merely antimicrobials but are highly effective resistance-modifying agents. Their ability to synergize with antibiotics, particularly against challenging Gram-negative pathogens, presents a promising strategy to combat antibiotic resistance. To further develop and capitalize on this promise, however, more research on its activities in vivo is required.

### 2.4. Evaluation of Cytotoxic Activity

**Figure 2:**
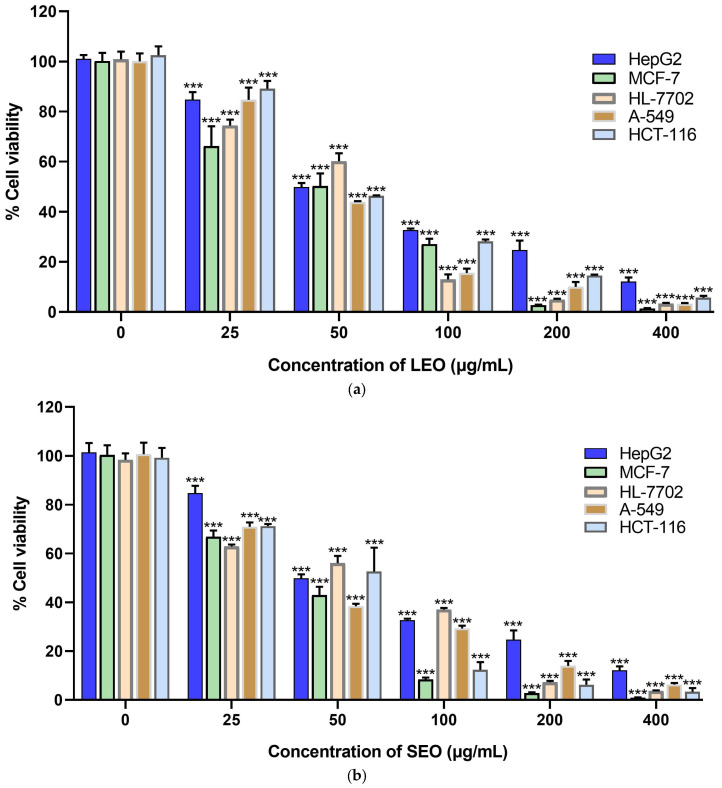

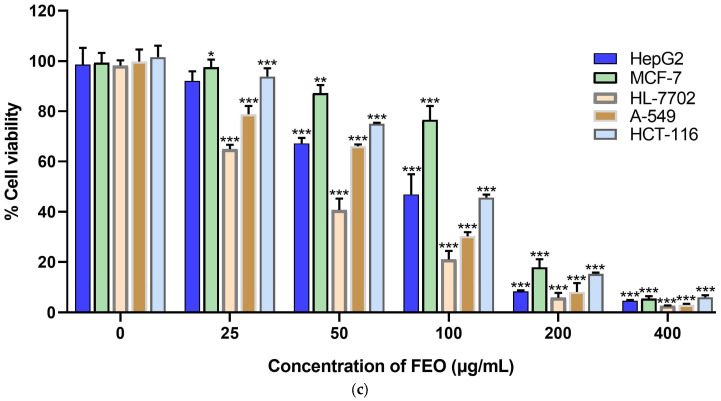
(**a**) Cytotoxic activity of LEO for 48 h; (**b**) cytotoxic activity of SEO for 48 h; (**c**) cytotoxic activity of FEO for 48 h. LEO: the essential oil of *R. bracteata* leaves; SEO: the essential oil of *R. bracteata* stems; FEO: the essential oil of *R. bracteata* flowers; *p*-values vs. untreated control of <0.05 (*), *p* < 0.01 (**), and *p* < 0.001 (***) were considered statistically significant differences.

The in vitro cytotoxic activities of the essential oils from the leaf (LEO), stem (SEO), and flower (FEO) of *Rosa bracteata* were evaluated by means of MTT assay against a panel of four human cancer cell lines—A549 (lung carcinoma), MCF-7 (breast adenocarcinoma), HepG2 (hepatocellular carcinoma), and HCT-116 (colorectal carcinoma)—and one normal human liver cell line (HL-7702). The activity is expressed as the half-maximal inhibitory concentration (IC_50_ in μg/mL, mean ± SD), with a lower value indicating higher cytotoxicity (Table 5). As shown in Figure 2, the *Rosa bracteata* essential oils displayed a dose-dependent cytotoxic activity against all tested cell lines. The stem essential oil (SEO) demonstrated the strongest cytotoxicity across all four cancer cell lines, showing the lowest IC_50_ values. For both LEO and SEO, the MCF-7 breast cancer cell line was the most sensitive, with the lowest IC_50_ values (LEO: 44.25 ± 5.74 μg/mL; SEO: 37.91 ± 2.10 μg/mL). The flower essential oil (FEO) was consistently the least cytotoxic against all cancer lines, with notably weak activity against MCF-7 cells (IC_50_ = 127.93 ± 9.66 μg/mL). While LEO and SEO showed similar cytotoxicity to HL-7702 as to some cancer lines, FEO was most toxic to the normal cells, exhibiting its lowest IC_50_ value (38.53 ± 0.55 μg/mL) against them.

The selective and enhanced cytotoxicity of stem essential oil (SEO) represents an interesting finding. Its stronger activity against all cancer cell lines compared to the other essential oils tested can be attributed to its chemical composition. SEO is dominated by a combination of recognized bioactive terpenes: (*E*)-nerolidol, phytol, and *α*-cadinol. These compounds have been individually reported to induce apoptosis and cell cycle arrest in various cancer models through mechanisms such as oxidative stress generation and mitochondrial dysfunction [57,58,59,60,61,62,63]. Meanwhile, the presence of benzyl benzoate may act as a key synergist, enhancing the penetration and efficacy of the other terpenes, thereby increasing the potency of SEO [64]. It is important to note that the MTT assay specifically reflects cellular metabolic activity, which can be influenced by both cell proliferation and cytotoxicity. To provide a more comprehensive assessment of cell viability, future studies would benefit from complementing this data with membrane integrity-based assays, such as the Neutral Red uptake or LDH release assays.

### 2.5. Evaluation of Antioxidant Activity

The antioxidant potential of the essential oils from leaves (LEO), stems (SEO), and flowers (FEO) of *Rosa bracteata* was evaluated using three distinct in vitro assays: 2,2-diphenyl-1-picrylhydrazyl (DPPH), 2,2′-azino-bis(3-ethylbenzothiazoline-6-sulfonic acid) (ABTS), and ferric-reducing antioxidant potential (FRAP). The results are summarized in Table 6. The data indicated a consistent trend across all three assays. The leaf essential oil (LEO) exhibited the strongest antioxidant activity, demonstrated by the lowest IC_50_ values in both the DPPH radical scavenging assay of (1.13 ± 0.12) × 10^3^ μg/mL and the ABTS radical scavenging assay of (0.285 ± 0.03) × 10^3^ μg/mL, and the highest reducing power in the FRAP assay (97.66 ± 11.27 μmol Trolox·g^−1^). The stem essential oil (SEO) showed intermediate activity, while the flower essential oil (FEO) displayed the weakest antioxidant capacity. However, compared with the reference standards Trolox (IC_50_ = 5.19 ± 0.56 μg/mL) and BHT (IC_50_ = 6.18 ± 0.39 μg/mL), all of the tested EOs possessed poor radical scavenging activities.

This antioxidant activity could be directly correlated to the unique chemical profile of EOs. The presence of several known antioxidant compounds, notably (*E*)-nerolidol and phytol, is likely a primary factor. Both sesquiterpenes (like nerolidol) and diterpenes (like phytol) have been previously reported to exhibit significant radical scavenging and reducing activities due to their chemical structure, which can donate hydrogen atoms or electrons to stabilize free radicals [40,65,66,67]. Furthermore, *α*-cadinol, a sesquiterpene alcohol present in all three oils at different concentrations, also contributes to antioxidant activity [68]. The presence of hexadecanoic acid (palmitic acid) may also play a synergistic role, as some fatty acids can influence the bioavailability and interaction of other antioxidant compounds [69]. While SEO shares important antioxidants with LEO, including (*E*)-nerolidol, phytol, and *α*-cadinol, its overall efficacy may be influenced by the presence of other major constituents like benzyl benzoate and (2*E*)-2-hexenyl benzoate [70]. These ester compounds are generally less recognized for potent antioxidant activity compared to alcohols like nerolidol and phytol [71]. Therefore, the ratio of highly active compounds to less active ones likely resulted in the intermediate antioxidant performance observed. The lower antioxidant activity of FEO can be explained by its distinctly different chemical composition. The flower essential oil is dominated by ethyl octanoate, octanoic acid, and dodecanoic acid. While some medium-chain fatty acids show mild antioxidant properties, they are typically far less effective than the terpenoid alcohols that dominate the leaf and stem oils [72].

## 3. Materials and Methods

### 3.1. Chemicals and Reagents

All chemicals and reagents used in this study were of analytical grade or higher purity. Anhydrous sodium sulfate (Na_2_SO_4_), methanol, dimethyl sulfoxide (DMSO), 2,2′-azino-bis(3-ethylbenzothiazoline-6-sulfonic acid) diammonium salt (ABTS), 2,2-diphenyl-1-picrylhydrazyl (DPPH), butylated hydroxytoluene (BHT), Trolox (6-hydroxy-2,5,7,8-tetramethylchroman-2-carboxylic acid), penicillin, chloramphenicol, streptomycin, doxorubicin, 3-(4,5-Dimethyl-2-thiazolyl)-2,5-diphenyl-2H-tetrazolium bromide (MTT), and the homologous series of *n*-hexane (C_7_-C_30_) were purchased from Sigma, St. Louis, MO, USA. 2, 4, 6-Tri (2-pyridyl)-s-triazine (TPTZ) was purchased from MERK, Darmstadt, Germany. RPMI 1640 medium, fetal bovine serum (FBS), and Mueller–Hinton broth were purchased from Thermo Fisher Scientific, Waltham, MA, USA. The tested cell lines were obtained from the Shanghai Institute for Biological Sciences (SIBS, Shanghai, China).

### 3.2. Plant Material

The materials of *R. bracteata* were collected from Lishui, Zhejiang Province, China (28°36′58.652″ N 120°3′5.574″ E), in August 2022. The plant specimens (No. 022032) were identified by Professor Hong Zhao and deposited in the Herbarium of Shandong University, China.

### 3.3. Essential Oil Extraction

Essential oil isolation from fresh leaves (0.7 kg), stems (0.9 kg), and flowers (1.0 kg) was performed separately through hydrodistillation for a period of four hours, employing a Clevenger-type apparatus. The process was carried out in three batches. The average yield of the essential oil was calculated based on the dry weight of the plant material. The extracted oils were collected, dried over anhydrous sodium sulfate, and then stored at 4 °C until further analysis. To assess in vitro biological efficacy, both the essential oils and standard reference drugs were initially dissolved in DMSO. A series of subsequent dilutions were then prepared using an appropriate culture medium, ensuring the final DMSO concentration did not exceed 1%.

### 3.4. Identification of EO Components

Chemical profiling of the essential oils (EOs) was conducted using an Agilent 7890A gas chromatograph system, fitted with an HP-5MS capillary column (30 m × 0.25 mm, 0.25 μm film thickness) and equipped with both an Agilent 5975C mass selective detector and a flame ionization detector (FID). Helium served as the carrier gas, set at a constant flow rate of 1.3 mL/min. The operational parameters for the GC-MS and GC-FID analyses followed previously established protocols [73]. Constituent identification was achieved by matching the acquired mass spectra against those contained in the NIST 14 and Wiley 10 databases, as well as with published spectral data [36,37,38], and comparing the measured retention indices (RI), calibrated against a series of *n*-alkanes (C_7_–C_30_), with literature values. The relative percentage of each component was determined from the GC-FID peak areas, utilizing a normalization method without correction factors.

### 3.5. Antibacterial Activity Assays

The antibacterial activity of the essential oils (EOs) was evaluated via the broth microdilution method [74]. The microbial strains investigated included *P. aeruginosa*, *E. coli*, *B. subtilis*, and *S. aureus*. The reference drug was chloramphenicol. In a 96-well microplate, the EOs underwent a two-fold serial dilution process using MH broth, with each well receiving 100 µL of the diluted sample (0.01 to 5.12 mg/mL). To this, 100 µL of a bacterial suspension, adjusted to a concentration of 10^6^ CFU/mL, was added. Following incubation at 37 °C for 24 h, bacterial growth was assessed using TTC as an indicator. The negative control, containing inoculated media without essential oil, verified robust microbial growth, while the blank control, containing sterile broth without oil or microbes, confirmed no contamination occurred. The minimum inhibitory concentration (MIC) was defined as the lowest sample concentration that prevented visible growth. To determine the minimum bactericidal concentration (MBC), a 100 µL subculture from wells showing no growth was inoculated and incubated again at 37 °C for 24 h.

### 3.6. Synergistic Effect Evaluation

To evaluate potential synergistic interactions, the micro broth checkerboard assay [75] was employed to test combinations of the essential oils (EOs) with either chloramphenicol or streptomycin. The concentrations of each sample were prepared at varying ranges from 4× to 1/32× their predetermined MIC values. Briefly, a 50 μL aliquot of each two-fold serially diluted EO was dispensed into specific wells of a 96-well plate, which contained 50 μL of the antibiotic at various concentrations and 100 μL of a bacterial suspension standardized to 10^6^ CFU/mL. The plates were subsequently incubated at 37 °C for a period of 24 h. Following incubation, the MICs for each agent, both alone and in combination, were recorded. The fractional inhibitory concentration index (FICI) was then calculated to quantify synergistic effects according to the formula:

(1) $$\mathrm{FICI}\;=\;\frac{\mathrm{MIC}\;\mathrm{of}\;\mathrm{EO}\;\mathrm{in}\;\mathrm{combination}}{\mathrm{MIC}\;\mathrm{of}\;\mathrm{EO}\;\mathrm{alone}}+\frac{\mathrm{MIC}\;\mathrm{of}\;\mathrm{antibiotic}\;\mathrm{in}\;\mathrm{combination}}{\mathrm{MIC}\;\mathrm{of}\;\mathrm{antibiotic}\;\mathrm{alone}}$$

Synergy is considered to exist if FICI ≤ 0.5 [45].

### 3.7. Cytotoxic Activity Evaluation

The cytotoxicity of the essential oil (EO) against four human cancer cell lines—MCF-7, A-549, HCT-116, and HepG2—along with the non-cancerous HL-7702 line, was assessed via MTT assay according to an established protocol [76]. Doxorubicin was tested as a reference. Cells were maintained in RPMI-1640 medium containing 10% fetal bovine serum (FBS), 2 mM glutamine, 100 U/mL penicillin, and 100 µg/mL streptomycin, under a 5% CO_2_ atmosphere at 37 °C. For the assay, 5 × 10^4^ cells per well were seeded into a 96-well plate using 100 µL of the complete RPMI medium with 10% FBS. After a 24-h incubation period, the EO was serially diluted to final concentrations ranging from 25.00 to 400.00 μg/mL and introduced to the wells. Forty-eight hours later, 20 µL of MTT solution (5 mg/mL) was added to each well, and the plates were incubated for another 4 h. The formazan crystals formed were then dissolved using 150 µL of dimethyl sulfoxide (DMSO). Absorbance was measured at a wavelength of 570 nm using a microplate reader (Epoch BioTek, Woodlands, TX, USA).

### 3.8. Antioxidant Activity Evaluation

#### 3.8.1. DPPH Radical Scavenging

The DPPH radical scavenging assay was performed using a 96-well plate, following the methodology outlined in reference [77]. Into each well, 150 µL of a 2,2-diphenyl-1-picrylhydrazyl radical solution (0.05 mg/mL) was introduced. A volume of 50 µL of the sample, prepared at a range of concentrations in methanol (0.05 to 3.2 mg/mL), was then added to the respective wells, with the exception of the blank control wells. For the control test, 200 µL of methanol was used instead. Following gentle shaking for one minute, the plate was kept in darkness for 6 h. Absorbance was measured at a wavelength of 517 nm using a microplate reader (Epoch BioTek, USA). The standard antioxidants BHT and Trolox were employed as reference compounds. The percentage of radical inhibition (I%) was determined using the following formula:

I (%) = [1 − (A_sample_ − A_blank_)/A_blank_)] × 100.

A represents the absorbance.

#### 3.8.2. ABTS Radical Cation Scavenging

The ABTS radical cation scavenging assay was conducted based on a reported method, with slight modifications [78]. Briefly, a 7 mM 2,2′-azino-bis(3-ethylbenzothiazoline-6-sulfonic acid) solution was reacted with 2.45 mM potassium persulfate to generate the ABTS^•+^ radical cation, which was then stored in darkness for 16 h. This resulting solution was subsequently diluted with phosphate-buffered saline (PBS) until an absorbance of 0.700 (±0.02) was achieved at 734 nm. The essential oil (EO) was dissolved in methanol to prepare a range of specific concentrations (0.05 to 3.2 mg/mL). For the test, 50 µL of each sample solution was added to 150 µL of the diluted ABTS^•+^ reagent within a 96-well plate. The combined mixture was incubated in the absence of light for 30 min, after which the absorbance was measured at 734 nm using a microplate reader (Epoch BioTek, USA). The scavenging capacity was calculated employing the same formula applied in the DPPH assay.

#### 3.8.3. Ferric Reducing Power

The ferric-reducing antioxidant potential (FRAP) assay was performed following a previously described method with slight modifications [79]. A FRAP reagent was prepared fresh by mixing 300 mM acetate buffer (pH 3.6), 10 mM TPTZ (2,4,6-tripyridyl-*S*-triazine) in 40 mM HCl, and 20 mM FeCl_3_·6H_2_O in a 10:1:1 ratio. To obtain a linear dose-response curve of Trolox, a stock solution of 2 mM of Trolox was prepared in methanol, from which 5 concentrations were prepared, including 400, 300, 200, 100, and 50 μM. In the wells of a 96-well plate, 20 µL of serially diluted EO (0.05 to 3.2 mg/mL) samples were added to 180 µL of the freshly prepared FRAP reagent. This mixture was incubated in darkness at 37 °C for a duration of 30 min. Following incubation, the absorbance was measured at 593 nm using a microplate reader (Epoch BioTek, USA). The results were quantified and expressed as Trolox equivalent antioxidant capacity.

### 3.9. Statistical Analysis

All of the assays were performed with three replicates, and the results are expressed as the mean ± standard deviation. For statistical analysis, IBM SPSS Statistics software (version 29) was employed. One-way ANOVA was used to test the statistical significance of the data, followed by Dunnett’s test. Differences from the control were considered significant when the *p*-values were lower than 0.05. Graphs were obtained using GraphPad Prism 8.

## 4. Conclusions

The study of the chemical composition of different parts of *R. bracteata* essential oils revealed notable variations among leaf (LEO), stem (SEO), and flower (FEO) oils. The stem oil was characterized by high concentrations of terpenoid alcohols and esters, notably (E)-nerolidol, phytol, and benzyl benzoate, while the flower oil was dominated by aliphatic compounds, including ethyl octanoate and octanoic acid. In contrast, the leaf oil presented a more balanced composition of sesquiterpenes, such as *α*-cadinol and (*E*)-nerolidol, alongside significant amounts of the alkene 1-pentadecene and fatty acids like hexadecanoic acid. The essential oils obtained from *R. bracteata* showed significant concentration-dependent antioxidant activity in ABTS and FRAP assays, with LEO demonstrating the strongest activity but relatively weaker DPPH radical scavenging capacity. Furthermore, the results of cytotoxicity assays indicated that SEO exhibited remarkable selective cytotoxic activities against MCF-7, A549, and HCT-116 cell lines. In addition, the evaluated essential oils demonstrated greater antibacterial efficacy against Gram-positive bacteria compared to Gram-negative strains. Among them, the stem-derived essential oil displayed the most potent antibacterial activity. A significant novel finding is the observation of strong synergistic effects between *R. bracteata* essential oils and the antibiotics chloramphenicol and streptomycin. This effect was especially pronounced against Gram-negative pathogens. Nevertheless, the exact mechanism of action under physiological conditions and its potential for clinical use require thorough investigation.

## Figures and Tables

**Table 1:** Chemical composition of the essential oils of leaf, stem, and flower of *R. bracteata*.

No.	R.T.	Compound	RI ^a^	RI ^b^	% Leaves	% Stems	% Flowers
**1**	4.60	Hexanoic acid	980	967			3.7
**2**	5.61	Phenyl acetaldehyde	1042	1046			0.6
**3**	4.80	(*E*)-2-Octenal	1053	1049	1.9		
**4**	6.02	1-Octanol	1067	1063			1.4
**5**	6.59	Linalool	1101	1095	1.2	2.6	
**6**	8.14	*α*-Terpineol	1192	1188		2.2	
**7**	8.20	Methyl salicylate	1195	1191	2.2	2.6	
**8**	8.20	Ethyl octanoate	1195	1196			31.4
**9**	8.43	Octanoic acid	1209	1202			25.8
**10**	8.69	8,9-Dehydrothymol	1224	1221			0.6
**11**	8.81	Isobornyl formate	1231	1235			0.5
**12**	8.90	(*Z*)-3-Hexenyl isovalerate	1236	1237		0.9	
**13**	9.33	(*Z*)-Chrysanthenyl acetate	1261	1264		1.7	1.5
**14**	9.50	Nonanoic acid	1271	1267			0.3
**15**	10.23	(*E*,*E*)-2,4-Decadienal	1315	1315	0.6		0.4
**16**	10.89	Dehydro-*ar*-ionene	1355	1355			0.3
**17**	11.06	Decanoic acid	1366	1364			0.6
**18**	11.28	8-Hydroxylinalool	1379	1367		0.8	
**19**	11.38	Butyl caprylate	1385	1393			0.4
**20**	11.40	(*E*)-2-Hexenyl hexanoate	1386	1391		1.0	
**21**	11.68	*α*-Gurjunene	1404	1409	2.0		
**22**	12.26	Thujopsene	1441	1441	0.3		
**23**	12.42	Geranyl acetone	1451	1453	0.4		0.1
**24**	12.60	Cabreuva oxide B	1463	1464		0.8	
**25**	12.85	*α*-Amorphene	1479	1483	0.8	2.0	0.4
**26**	13.00	1-Pentadecene	1489	1489	10.6		0.6
**27**	13.15	Benzyl tiglate	1499	1497	0.5	0.7	
**28**	13.21	*β*-Guaiene	1502	1502		0.7	
**29**	13.29	Tridecanal	1508	1509	0.4		0.6
**30**	13.36	1,1,4,5,6-Pentamethyl-Indan	1513	1523			0.3
**31**	13.55	*δ*-Cadinene	1526	1522			0.3
**32**	13.70	*α*-Copaen-11-ol	1535	1539	0.9		
**33**	14.10	(*E*)-Nerolidol	1562	1561	7.1	18.5	
**34**	14.17	Dodecanoic acid	1567	1565			4.4
**35**	14.23	(*Z*)-3-Hexen-1-ol benzoate	1571	1573	1.5	3.7	
**36**	14.27	Caryophyllene oxide	1574	1582		1.2	
**37**	14.32	Hexyl benzoate	1577	1579	0.9	1.7	1.0
**38**	14.43	(2*E*)-2-Hexenyl benzoate	1585	1587	1.8	5.5	
**39**	14.47	Isoaromadendrene epoxide	1588	1594		1.8	
**40**	14.52	Ethyl dodecanoate	1591	1594			1.2
**41**	14.61	Viridiflorol	1597	1592	0.8	1.5	
**42**	14.79	Cedrol	1609	1600	1.0	1.1	0.3
**43**	14.86	(*E*)-Longipinocarveol	1615	1618		1.9	
**44**	15.01	Junenol	1625	1619		0.8	
**45**	15.07	Selin-6-en-4α-ol	1630	1636		1.3	
**46**	15.29	epi-*α*-Cadinol	1645	1640	0.3	2.0	0.4
**47**	15.50	*α*-Cadinol	1660	1665	10.4	4.9	10.4
**48**	15.59	(*Z*)-7-Tetradecen-1-ol	1666	1660	13.9		
**49**	15.67	*α*-Bisabolol	1672	1673	1.2	3.6	0.8
**50**	15.76	Cadalene	1679	1676		0.9	
**51**	15.89	1-Heptadecene	1687	1687	3.4		
**52**	16.20	1-Pentadecanal	1710	1715	0.6	1.6	0.3
**53**	16.74	*α*-Bisabolol oxide A	1751	1748			0.2
**54**	16.83	Tetradecanoic acid	1758	1758	1.2		1.3
**55**	16.95	Benzyl Benzoate	1767	1760	1.5	9.0	
**56**	17.05	Octyl octanoate	1774	1779			0.6
**57**	17.06	(*Z*)-9-Hexadecenal	1775	1759	0.4		0.7
**58**	17.25	Ethyl tetradecanoate	1789	1795			0.2
**59**	17.92	Hexahydrofarnesyl acetone	1841	1845	3.1	3.0	0.3
**60**	18.09	*Z*-9-Hexadecen-1-ol	1854	1863	1.2		0.3
**61**	18.36	1-Hexadecanol	1876	1874			0.5
**62**	18.92	Methyl hexadecanoate	1920	1921			0.2
**63**	19.20	Isophytol	1943	1946	1.1	0.5	
**64**	19.41	Hexadecanoic acid	1961	1959	8.0	2.1	2.5
**65**	19.75	Ethyl hexadecanoate	1989	1992			1.0
**66**	19.82	Panaxjapyne A	1994	1994	2.7		
**67**	19.92	Hexadecyl acetate	2003	2003			0.2
**68**	20.20	Geranyl linallol	2027	2020	2.6	0.6	
**69**	20.81	Methyl linoleate	2079	2085	0.6	0.7	
**70**	21.03	*γ*-Palmitolactone	2098	2104	0.8	0.9	0.3
**71**	21.15	Phytol	2109	2114	6.9	9.1	0.2
**72**	21.43	Linoleic acid	2134	2132			0.3
**73**	21.47	Oleic Acid	2138	2141	1.5	2.0	
Total identified					96.3	95.9	97.4

^a^ Retention index calculated from n-alkanes (C_7_–C_30_) on an HP-5MS column; ^b^ Retention index data from the literature [36,37,38]; classes of compounds: oxygenated monoterpenes (No. **5**–**6**, **10**, **11**, **13**, **18**, **23**), sesquiterpene hydrocarbons: (No. **16**, **21**, **22**, **25**, **28**, **31**, **50**), oxygenated sesquiterpenes (No. **24**, **32**, **33**, **36**, **39**, **41**–**47**, **49**, **53**, **59**), oxygenated diterpenes (No. **63**, **68**, **71**), fatty acids and derivatives (No. **1**, **7**–**9**, **12**, **14**, **17**, **19**, **20**, **27**, **34**, **35**, **37**, **38**, **40**, **54**–**56**, **58**, **62**, **64**, **65**, **67**, **69**, **70**, **72**, **73**), carbonylic compounds (No. **2**, **3**, **15**, **29**, **52**, **57**), and others (No. **4**, **26**, **30**, **48**, **51**, **60**, **61**, **66**).

**Table 2:** Antibacterial activity of essential oil from different parts of *R. bracteata*.

Microorganism		MIC (μg/mL)				MBC (μg/mL)		
	LEO	SEO	FEO	Chl	LEO	SEO	FEO	Chl
Gram positive								
*B. subtilis* ATCC 6633	80.00	40.00	80.00	2.00	80.00	80.00	80.00	2.00
*S. aureus* ATCC 6538	160.00	80.00	160.00	2.00	160.00	160.00	160.00	16.00
Gram negative								
*E. coli* ATCC 25922	320.00	160.00	640.00	8.00	640.00	320.00	640.00	32.00
*P. aeruginosa* ATCC 27853	320.00	320.00	640.00	128.00	640.00	1280.00	1280.00	256.00

Chl: Chloramphenicol (Chl) was included as a positive control; MIC: Minimal inhibitory concentration; MBC: Minimal bactericidal concentration; LEO: the essential oil of *R. bracteata* leaves; SEO: the essential oil of *R. bracteate* stems; FEO: the essential oil of *R. bracteata* flowers. The data are the consensus of the three biological replicates.

**Table 3:** Fractional inhibitory concentration indices (FICIs) of chloramphenicol combined with *R. bracteata* essential oils against tested bacterial strains.

Strains	Sample	MICa (μg/mL)	MICc (μg/mL)	FICI
*Bacillus subtilis*	LEO	80.00	20.00	0.50 (S)
	Chl	2.00	0.50	
	SEO	40.00	10	0.38 (S)
	Chl	2.00	0.25	
	FEO	80.00	20.00	0.38 (S)
	Chl	2.00	0.25	
*Staphylococcus aureus*	LEO	160.00	40.00	0.38 (S)
	Chl	2.00	0.25	
	SEO	80.00	20	0.38 (S)
	Chl	2.00	0.25	
	FEO	160.00	40.00	0.50 (S)
	Chl	2.00	0.50	
*Escherichia coli*	LEO	320.00	20.00	0.08 (S)
	Chl	8.00	0.13	
	SEO	160.00	5.00	0.06 (S)
	Chl	8.00	0.25	
	FEO	640.00	160.00	0.38 (S)
	Chl	8.00	1.00	
*Pseudomonas aeruginosa*	LEO	320.00	20.00	0.07 (S)
	Chl	128.00	0.50	
	SEO	320.00	20.00	0.06 (S)
	Chl	128.00	0.25	
	FEO	640.00	40.00	0.13 (S)
	Chl	128.00	8.00	

MICa: MIC alone; MICc: MIC combined; Chl: chloramphenicol; LEO: the essential oil of *R. bracteata* leaves; SEO: the essential oil of *R. bracteata* stems; FEO: the essential oil of *R. bracteata* flowers; S: synergy. The data were determined in biological triplicate.

**Table 4:** Fractional inhibitory concentration indices (FICIs) of streptomycin combined with *R. bracteata* essential oils against tested bacterial strains.

Strains	Sample	MICa (μg/mL)	MICc (μg/mL)	FICI
*Bacillus subtilis*	LEO	80.00	10.00	0.38 (S)
	SM	1.00	0.25	
	SEO	40.00	5.00	0.26 (S)
	SM	1.00	0.13	
	FEO	80.00	10.00	0.38 (S)
	SM	1.00	0.25	
*Staphylococcus aureus*	LEO	160.00	20.00	0.38 (S)
	SM	1.00	0.25	
	SEO	80.00	10.00	0.26 (S)
	SM	1.00	0.13	
	FEO	160.00	40.00	0.50 (S)
	SM	1.00	0.25	
*Escherichia coli*	LEO	320.00	10.00	0.10 (S)
	SM	2.00	0.13	
	SEO	160.00	2.50	0.08 (S)
	SM	2.00	0.13	
	FEO	640.00	40.00	0.31 (S)
	SM	2.00	0.50	
*Pseudomonas aeruginosa*	LEO	320.00	20.00	0.13 (S)
	SM	4.00	0.25	
	SEO	320.00	20.00	0.10 (S)
	SM	4.00	0.13	
	FEO	640.00	20.00	0.09 (S)
	SM	4.00	0.25	

MICa: MIC alone; MICc: MIC combined; SM: streptomycin; LEO: the essential oil of *R. bracteata* leaves; SEO: the essential oil of *R. bracteata* stems; FEO: the essential oil of *R. bracteata* flowers; S: synergy. The data were determined in biological triplicate.

**Table 5:** Results of cytotoxic activity (IC_50_ μg/mL) of *R. bracteata* essential oils.

	A549	MCF-7	HepG2	HCT-116	HL-7702
LEO	49.51 ± 2.25	44.25 ± 5.74	66.55 ± 0.32	59.60 ± 1.50	49.15 ± 2.97
LEO-SI	0.99	1.11	0.74	0.82	
SEO	43.91 ± 1.36	37.91 ± 2.10	51.15 ± 6.42	44.76 ± 4.49	49.01 ± 1.46
SEO-SI	1.12	1.30	0.96	1.10	
FEO	61.57 ± 2.91	127.93 ± 9.66	79.80 ± 3.01	90.10 ± 1.22	38.53 ± 0.55
FEO-SI	0.63	0.30	0.48	0.43	
Doxorubicin	1.47 ± 0.09	1.36 ± 0.03	1.87 ± 0.13	0.99 ± 0.04	0.80 ± 0.04

LEO: the essential oil of *R. bracteata* leaves; SEO: the essential oil of *R. bracteata* stems; FEO: the essential oil of *R. bracteata* flowers; LEO-SI: Selectivity indices of LEO; SEO-SI: Selectivity indices of SEO; FEO-SI: Selectivity indices of FEO; doxorubicin was tested as a reference; data are expressed as the mean ± SD of triplicate experiments. Selectivity Index (SI) was calculated as IC_50_ on normal human liver cells (HL-7702) divided by IC_50_ on the respective cancer cell line. An SI > 1 indicates selective cytotoxicity towards the cancer cells.

**Table 6:** Results of antioxidant activity in vitro (DPPH, ABTS, and FRAP) of the essential oil from different parts of *R. bracteata*.

Test Sample	DPPH IC_50_ (μg/mL)	ABTS IC_50_ (μg/mL)	FRAP (μmol Trolox·g^−1^)
LEO	(1.13 ± 0.12) × 10^3^	(0.29 ± 0.03) × 10^3^	97.66 ± 11.27
SEO	(1.38 ± 0.16) × 10^3^	(0.38 ± 0.05) × 10^3^	79.52 ± 8.32
FEO	(1.43 ± 0.37) × 10^3^	(0.69 ± 0.06) × 10^3^	55.36 ± 6.38
BHT *	6.18 ± 0.39	4.16 ± 0.25	
Trolox *	5.19 ± 0.56	1.68 ± 0.17	

* Positive control used for DPPH and ABTS assays. For the FRAP assay, the results are quantified and expressed as Trolox Equivalent Antioxidant Capacity (TEAC). LEO: the essential oil of *R. bracteata* leaves; SEO: the essential oil of *R. bracteata* stems; FEO: the essential oil of *R. bracteata* flowers. Data are expressed as mean ± SD (*n* = 3).

## Data Availability

The data are contained in this article.
